# Supplementary material for: Biodegradation of benzo[a]pyrene by division of labor in co-culture of Bacillus haynesii and Kluyveromyces marxianus from kefir
Source: Microbiol Spectr. 2025 Nov 3;13(12):e01840-25. doi: 10.1128/spectrum.01840-25 (PMC12671072; doi:10.1128/spectrum.01840-25)
Supplement: Supplemental figures and tables — Figures S1 and S2; Tables S1 and S2. [file spectrum.01840-25-s0001.docx]

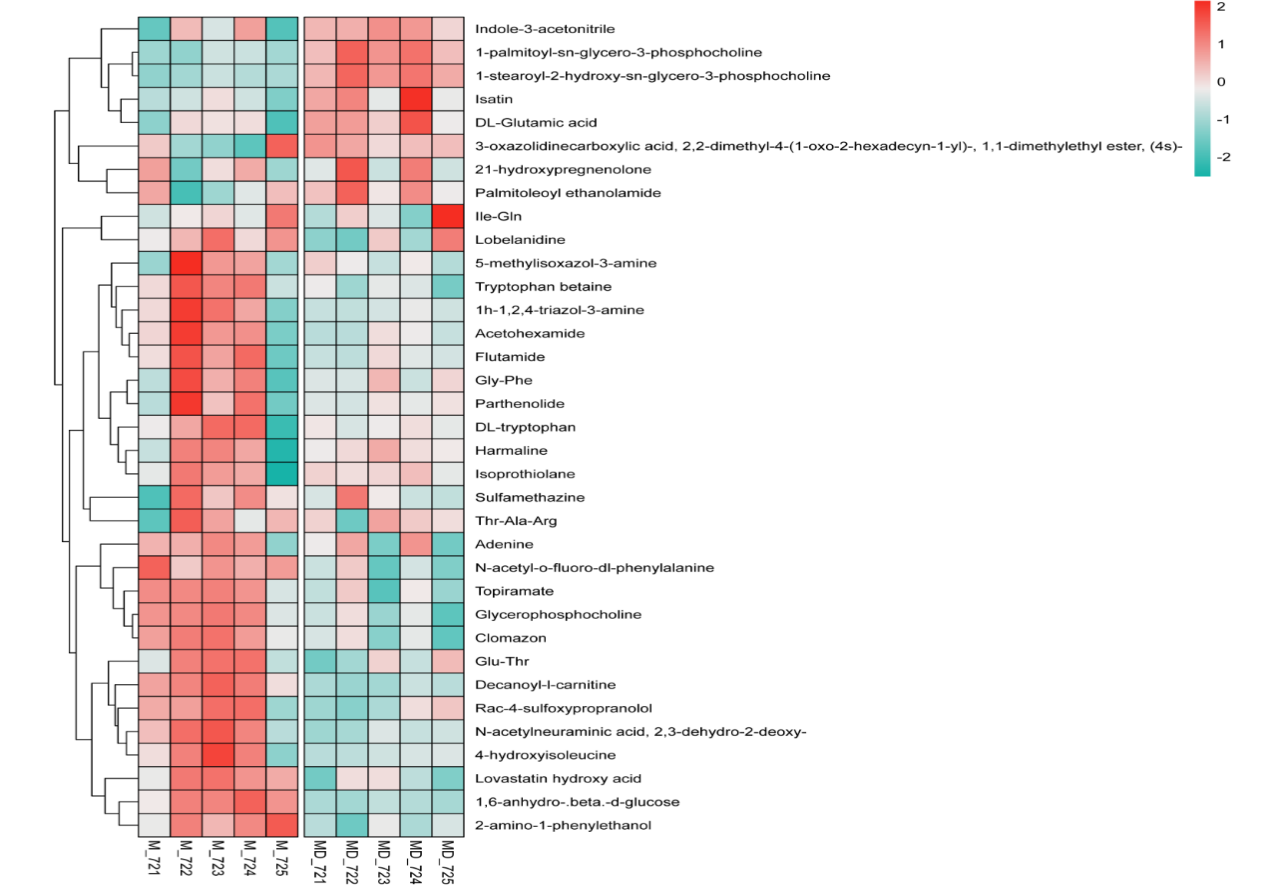


a

M72 vs MD72


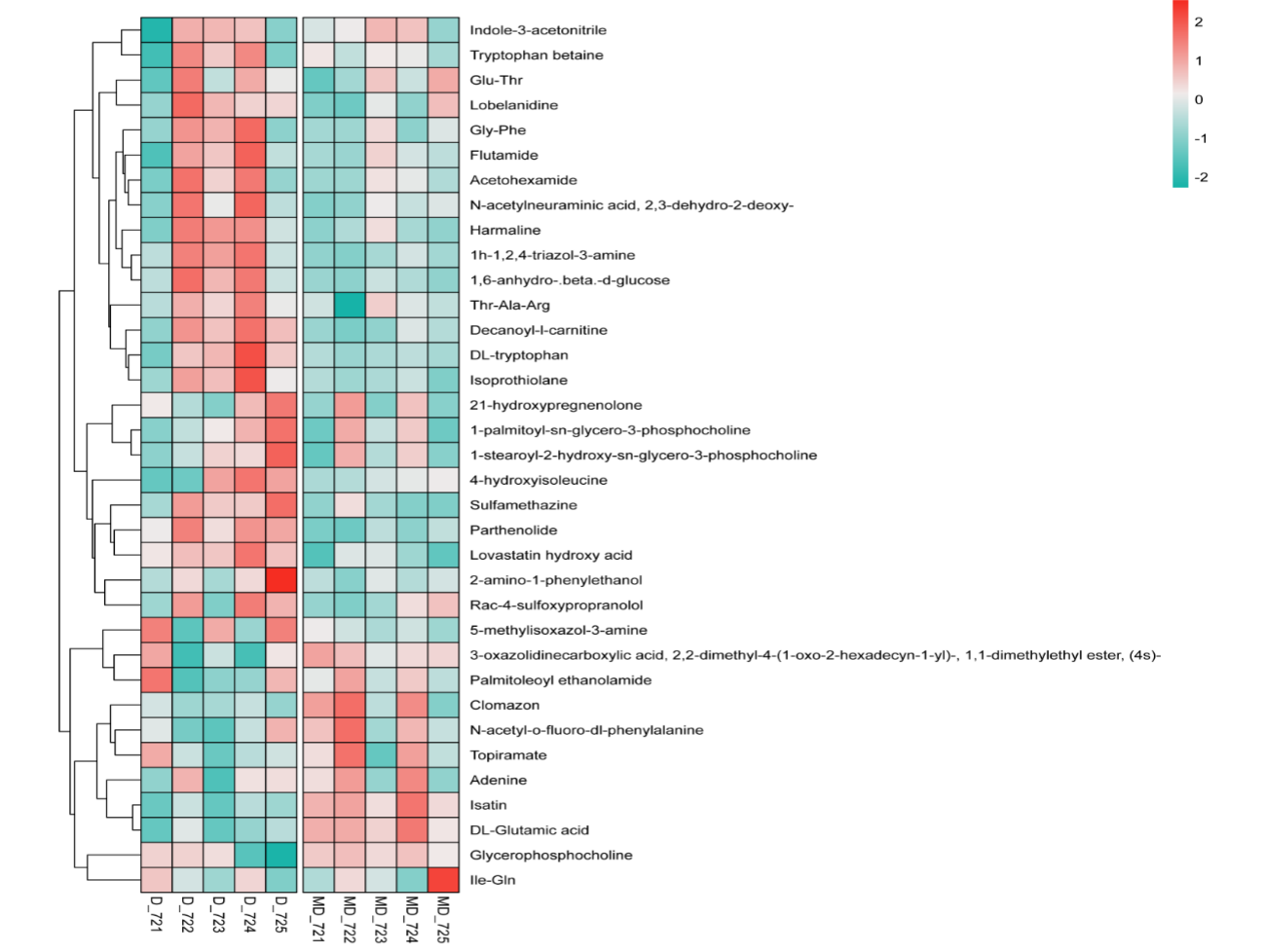


b

D72 vs MD72

Figure S1 The clustering tree diagram represents discriminating metabolites in each test group i.e., M72 vs MD72 (a), D72 vs MD72 (b). Every row denotes a metabolite, and every column a sample. Color intensity reflects the concentration of each metabolite (red for upregulated, green for downregulated). The co-culture metabolite data set was filtered using VIP > 1 and P < 0.05.


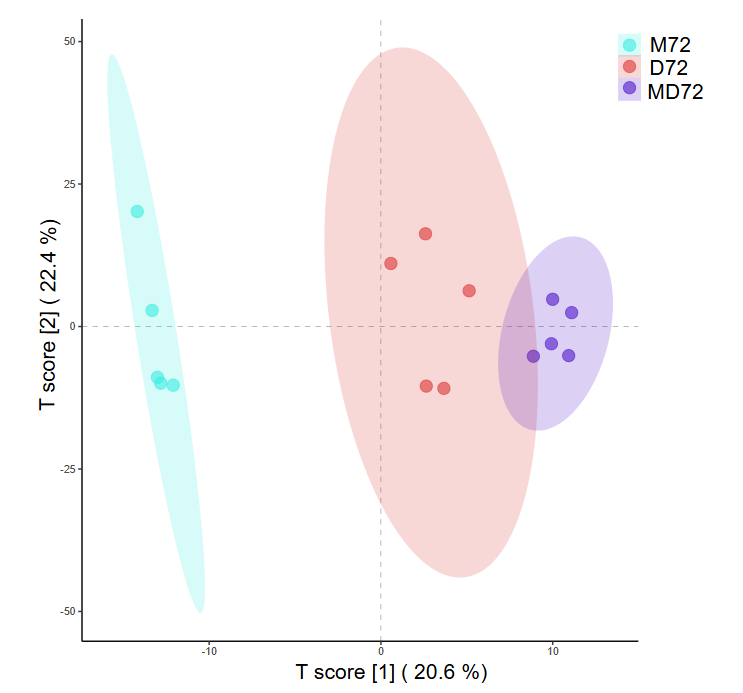


Figure S2 The Partial least squares discriminant analysis (OPLS-DA) score plot show a clear separation degree of the three groups of samples.

Table S1 Independent variables and their levels in Box-Behnken design.

| Independent variables | Symbols | Coded level | | |
| --- | --- | --- | --- | --- |
|  |  | -1 | 0 | 1 |
| Initial Bap concentration（mg/L） | A | 10 | 20 | 30 |
| Temperature (℃) | B | 27 | 32 | 37 |
| pH | C | 5 | 6 | 7 |
| Time（h） | D | 60 | 72 | 84 |

Table S2 Program of Box-Behnken experimental design and response value

| Number | A: Initial Bap concentration (mg/L) | B: Temperature (℃) | C: pH | D: Time (h) | Bap biodegradation rate (%) |
| --- | --- | --- | --- | --- | --- |
| 1 | 20.00 | 32 | 6.00 | 72.00 | 68.60 |
| 2 | 10.00 | 32 | 7.00 | 72.00 | 38.58 |
| 3 | 20.00 | 37 | 7.00 | 72.00 | 55.16 |
| 4 | 20.00 | 27 | 6.00 | 84.00 | 51.46 |
| 5 | 20.00 | 32 | 6.00 | 72.00 | 65.88 |
| 6 | 20.00 | 32 | 5.00 | 60.00 | 22.50 |
| 7 | 30.00 | 27 | 6.00 | 72.00 | 33.81 |
| 8 | 30.00 | 32 | 6.00 | 84.00 | 48.16 |
| 9 | 20.00 | 37 | 5.00 | 72.00 | 53.59 |
| 10 | 20.00 | 32 | 7.00 | 60.00 | 24.15 |
| 11 | 10.00 | 32 | 5.00 | 72.00 | 44.71 |
| 12 | 10.00 | 32 | 6.00 | 60.00 | 17.93 |
| 13 | 20.00 | 32 | 7.00 | 84.00 | 64.29 |
| 14 | 30.00 | 37 | 6.00 | 72.00 | 46.11 |
| 15 | 30.00 | 32 | 6.00 | 60.00 | 18.52 |
| 16 | 20.00 | 37 | 6.00 | 84.00 | 68.32 |
| 17 | 30.00 | 32 | 7.00 | 72.00 | 36.57 |
| 18 | 20.00 | 37 | 6.00 | 60.00 | 24.17 |
| 19 | 20.00 | 27 | 7.00 | 72.00 | 34.03 |
| 20 | 20.00 | 32 | 6.00 | 72.00 | 66.57 |
| 21 | 10.00 | 27 | 6.00 | 72.00 | 37.42 |
| 22 | 30.00 | 32 | 5.00 | 72.00 | 43.38 |
| 23 | 20.00 | 32 | 5.00 | 84.00 | 58.79 |
| 24 | 10.00 | 37 | 6.00 | 72.00 | 50.57 |
| 25 | 20.00 | 27 | 6.00 | 60.00 | 15.98 |
| 26 | 20.00 | 32 | 6.00 | 72.00 | 68.94 |
| 27 | 20.00 | 27 | 5.00 | 72.00 | 43.80 |
| 28 | 10.00 | 32 | 6.00 | 84.00 | 59.50 |
| 29 | 20.00 | 32 | 6.00 | 72.00 | 62.71 |
